# Novel proteome and acetylome of *Bemisia tabaci* Q in response to *Cardinium* infection

**DOI:** 10.1186/s12864-018-4907-3

**Published:** 2018-07-05

**Authors:** Hongran Li, James D. Harwood, Tongxian Liu, Dong Chu

**Affiliations:** Key Laboratory of Integrated Crop Pest Management of Shandong Province, College of Plant Health and Medicine, Qingdao Agricultural University, Qingdao, 266109 Shandong China

**Keywords:** *Bemisia tabaci* Q, *Cardinium*, Physiological response, Proteomes, Acetylomes

## Abstract

**Background:**

It has become increasingly clear that symbionts have crucial evolutionary and ecological ramifications for their host arthropods. However, little is known whether these symbiont infections influence the proteome and lysine acetylome of their host arthropods. Here we performed experiments to investigate the proteomes and acetylomes of *Cardinium*-infected (C^*+^) and -uninfected (C^−^) *Bemisia tabaci* Q with identical backgrounds, through the combination of affinity enrichment and high-resolution LC-MS/MS analysis.

**Results:**

Of the 3353 proteins whose levels were quantitated in proteome, a total of 146 proteins dividing into 77 up-regulated and 69 down-regulated proteins were discovered to be differentially expressed as having at least a 1.2-fold change when C^*+^ strain was compared with C^−^ strain. Furthermore, a total of 528 lysine acetylation sites in 283 protein groups were identified, among which 356 sites in 202 proteins were quantified. The comparison of acetylomes revealed 30 sites in 26 lysine acetylation proteins (Kac) were quantified as up-regulated targets and 35 sites in 29 Kac proteins were quantified as down-regulated targets. Functional analysis showed that these differentially expressed proteins and Kac proteins were mainly involved in diverse physiological processes related to development, immune responses and energy metabolism, such as retinol metabolism, methane metabolism and fatty acid degradation. Notably, protein interaction network analyses demonstrated widespread interactions modulated by protein acetylation.

**Conclusion:**

Here we show the proteome and acetylom of *B. tabaci* Q in response to the symbiont *Cardinium* infection. This is the first study to utilize the tool of acetylome analysis for revealing physiological responses of arthropods to its symbiont infection, which will provide an important resource for exploring the arthropod-symbiont interaction.

**Electronic supplementary material:**

The online version of this article (10.1186/s12864-018-4907-3) contains supplementary material, which is available to authorized users.

## Background

Symbiotic bacteria (symbionts) and insects commonly form intimate associations, and result from co-evolution and strictly vertical transmission. Many of these symbionts are mainly defined two categories: primary symbionts and facultative ones [1]. These primary symbionts can provide essential amino acids and carotenoids to insects by synthesizing essential amino acid, while facultative symbionts are not necessary for all hosts but may importantly affect the host biology and may confer resistance to pathogens, parasitoid wasps heat stress, and even act as a manipulator of the reproductive modes of its hosts [2–4]. As the development of sequencing technology and bioinformatics, huge genomic resources have greatly broadened our understanding for the molecular mechanisms of insect-symbiont interactions. However, proteomic studies in this field have reached relatively little attention.

The whitefly *Bemisia tabaci* (Gennadius) Q (commonly known as *B. tabaci* MED) is originated from Mediterranean but represents an exotic whitefly species in China [5]. Like most phloem-feeding insects, it has co-evolved with several bacterial symbionts that may play important roles in both ecological and biological processes [6, 7]. One of facultative symbionts, *Candidatus* Cardinium hertigii (henceforth referred to as *Cardinium*) was first characterized in *B. tabaci* by Weeks et al. (2003) [8]. Our long-term field research has previously demonstrated that the infection ratio of *Cardinium* in *B. tabaci* Q remains low (12.2%) in Shandong Province, China [9]. Laboratory experiments further revealed that the competitive ability and fitness of *Cardinium*-infected *B. tabaci* Q strain were weaker than these of uninfected strains [10]. In order to explore the underling mechanisms how *Cardinium* affects its host whitefly fitness, the genome-wide transcriptomes and small RNA of the two whitefly populations were analyzed using Illumina sequencing technology (unpublished data). In general, microarray technology and mRNA detection techniques often do not accurately reflect abundances of downstream effector proteins, thus the strategies focusing directly on the protein quantification or/and post-translational modification (PTM) were increasingly epidemic and effective [11–13].

There are accumulating evidences for using quantitative proteomics to exploit the central role of the resident symbiont in the physiology of insects, such as in *Acyrthosiphonpisum*, *Camponotuschromaiodes*, and *Drosophila melanogaster*) [14–16]. But rarely PTM research on insect-symbiont interactions was presented. PTMs can alter structural, conformational and physicochemical properties of proteins, which involved in many biological processes [17]. Among all the amino acids, lysine is a frequent target to be modified, which can be subjected to a variety of PTM [18]. Lysine acetylation is one reversible and highly conserved PTM that plays an essential role in a broad array of cellular physiological processes, including metabolic pathways, protein interactions and enzymatic activity [19–21]. With the advances in high-resolution mass spectrometry (MS) and antibody-based affinity enrichment of lysine residues, a lot of acetylomes have been identified in rats, *Drosophila melanogaster*, *Bombyx mori* and other species [22–26]. Here we document the physiological responses of an exotic whitefly in response to *Cardinium* infection through protein abundance and PTM analysis, illustrating a promising approach to study interactions between host insects and their symbionts.

In this study, we first established a *Cardinium*-infected *B. tabaci* Q strain (abbreviated as C^*+^) and an uninfected strain (abbreviated as C^−^) with identical genetic background. Then, we quantitatively examined proteomes and acetylomes in the two whitefly strains using a tandem mass tagging (TMT)-based quantitative proteomic method. This is the first study to utilize the tool of acetylomes for revealing the physiological responses of arthropods to symbiont infection, which will provide novel insights into these arthropod-symbiont interactions.

## Methods

### Whitefly colony and introgression

**Fig. 1:**
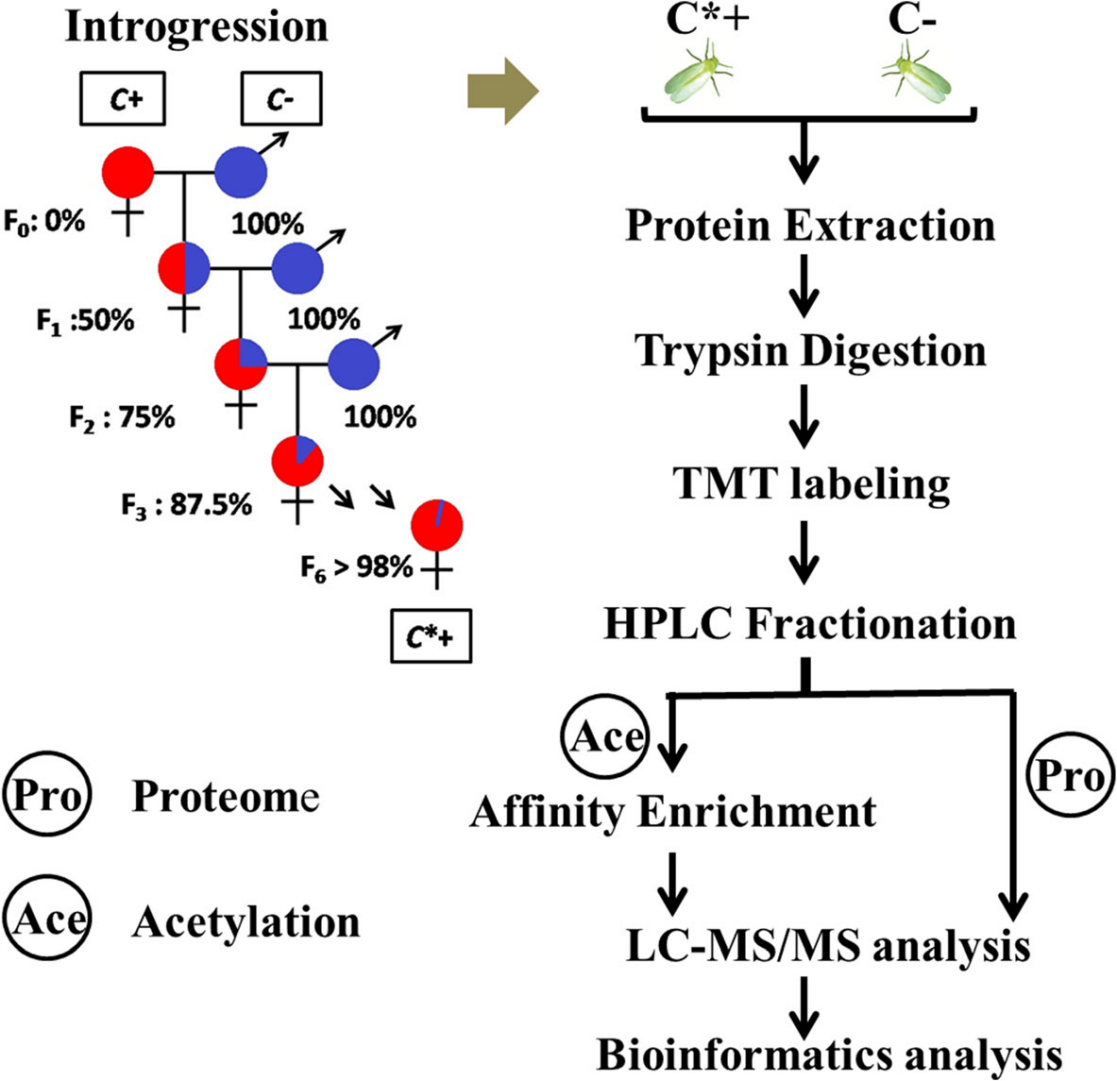
Experimental design and schematic diagram of the workflow used in this study. The whitefly photo was taken and processed by author Hongran Li

The *Cardinium* infected (C^+^) and uninfected (C^−^) *B. tabaci* Q populations used in this study were originally collected from cotton in Shandong Province, China, in July 2012, and maintained in separate cultures on potted cotton plants, Lu-Mian-Yan 21 cultivar (produced by Shandong Cotton Research Center, Jinan, Shandong, China) in isolated screen cages under controlled conditions (27 ± 1 °C with a 16/8 h light/dark photoperiod, RH 70 ± 5%). The introgressive backcrossing scheme was used to homogenize nuclear genetic backgrounds of infected and uninfected whiteflies, following the method described by Turelli and Hoffmann (1991) [27]. At first, about 30 *Cardinium*-uninfected males were collected to mate with a cohort of about 20 *Cardinum*-infected females to guarantee mated sufficiently. Then in subsequent generations, always *Cardinium*-uninfected males were mated to the introgressed *Cardinum*-infected female progeny for 6 generations. After completion of the introgression series, > 98% of nuclear alleles shared between C^*+^ and C^−^ whiteflies of the same line were regarded as two strains with identical genetic backgrounds in the present study (Fig. 1). Adult individuals of the *Cardinium*-infected and -uninfected *B. tabaci* Q strains with identical genetic backgrounds were stored at − 80 °C until required for protein preparation.

### *Cardinium* detection in populations

The symbiont *Cardinium* in each population of whitefly was identified every 30 days with a sample size of 20 adults, and detection was based on the amplification of the 16S rRNA gene (Additional file 1: Figure S1). The primers used for detection of *Cardinium* were CFB-F (5’-GCGGTGTAAAATGAGCGTG-3′) and CFB-R (5’-ACCTMTTCTTAACTCAAGCCT-3′) [8]. All PCRs were performed in 13 μL reaction volumes containing 1 × buffer, 0.16 mM of each dNTP, 0.5 mM of each primer, 0.5 unit Taq DNA polymerase (Takara) and 2 μL DNA. The negative control of each PCR consisted of sterile water substituting for DNA and the positive control of PCR was the use the *Cardinium*-infected whitefly DNA. Cycling conditions were as follows: initial denaturing at 95 °C for 5 min, followed by 35 cycles of 1 min at 94 °C for denaturation, 1 min at 58 °C for annealing, and 1 min at 72 °C for elongation, and the final extension at 72 °C for 7 min. All resultant PCR produces were electrophoresed with the negative control and positive control of the symbiont on a 1.5% agarose gel and visualized by *Gelview* staining.

### Protein extraction and digestion

Approximately 200 mg weighted adult individuals of the *B. tabaci* C^*+^ or C^−^ strains with mixed ages with ≈1-week post emergence were ground by liquid nitrogen, the resultant cell powder transferred to 5 mL centrifuge tube and sonicated three times on ice using a high intensity ultrasonic processor (Scientz) in lysis buffer (8 M urea, 2 mM EDTA, 10 mM DTT, and 1% Protease Inhibitor Cocktail). Samples were sonicated 38 times for 3 s with a 5 s break (20 KHz, 195 w). The remaining debris was removed by centrifugation at 20,000 g at 4 °C for 10 min. Finally, the protein was precipitated with cold 15% TCA for 2 h at − 20 °C. After further centrifugation at 4 °C for 10 min, the supernatant was discarded. The remaining precipitate was washed with cold acetone three times. The protein was re-dissolved in buffer (8 M urea, 100 mM TEAB, pH 8.0) and the protein concentration was determined with 2-D Quant kit (GE Healthcare) following the manufacturer’s instructions.

For digestion, the protein solution was reduced with 10 mM DTT for 1 h at 37 °C and alkylated with 20 mM IAA for 45 min at room temperature in the dark. For trypsin digestion, the protein sample was diluted by adding 100 mM TEAB to urea at a concentration less than 2 M. Finally, trypsin was added at a 1: 50 trypsin: protein mass ratio for the first digestion overnight and 1: 100 trypsin: protein mass ratio for a second 4 h-digestion. Approximately 100 μg protein for each sample was digested with trypsin for the following experiments.

### TMT labeling and HPLC fractionation

The peptide was desalted by a Strata X C18 SPE column (Phenomenex) and vacuum-dried. The peptide was reconstituted in 0.5 M TEAB and processed according to the manufacturer’s protocol for the 6-plex TMT kit. In summary, one unit of TMT reagent (defined as the amount of reagent required to label 100 μg of protein) was thawed and reconstituted in 24 μl ACN. The peptide mixtures were then incubated for 2 h at room temperature and pooled, desalted and dried by vacuum centrifugation.

The sample was then separated into fractions by high pH reverse-phase HPLC using an Agilent 300Extend C18 column (5 μm particles, 4.6 mm ID, 250 mm in length). To summarize the protocol, the peptides were first separated with a gradient of 2 to 60% acetonitrile in 10 mM ammonium bicarbonate pH 10 for 80 min into 80 fractions. Subsequently, the peptides were combined into 18 fractions and dried by vacuum centrifugation.

### Affinity enrichment of lysine acetylation peptides

To enrich lysine acetylation peptides, tryptic peptides dissolved in NETN buffer (100 mMNaCl, 1 mM EDTA, 50 mMTris-HCl, 0.5% NP-40, pH 8.0) were incubated with anti-acetyllysine antibody agarose conjugated beads ((PTM Biolabs, Hangzhou, China)) in a ratio of 15 μL beads/mg proteins at 4 °C overnight with gentle shaking. The beads were washed four times with NETN buffer and twice with ddH_2_O. The beads were washed four times with NETN buffer and twice with ddH_2_O. The bound peptides were eluted from the beads with 0.1% TFA. The eluted fractions were combined and vacuum-dried. The resultant peptides were cleaned with C18 Zip Tips (Millipore) according to the manufacturer’s instructions.

### LC-MS/MS measurement

Peptides were dissolved in 0.1% FA and directly loaded onto a reversed-phase pre-column (Acclaim PepMap 100, Thermo Scientific). Peptide separation was then performed using a reversed-phase analytical column (Acclaim PepMap RSLC, Thermo Fisher Scientific, Waltham, MA, USA). The gradient increased from 6 to 22% solvent B (0.1% FA in 98% ACN) over 22 min, 22 to 36% in 10 min and increasing to 85%. Finally, a holding phase at 85% for 5 min was performed, all at a constant flow rate of 400 nL/min on an EASY-nLC 1000 UPLC system. The resulting peptides were analyzed by Q Exactive™ plus hybrid quadrupole-Orbitrap mass spectrometer (Thermo Fisher Scientific, Waltham, MA, USA).

The peptides were subjected to NSI source followed by tandem mass spectrometry (MS/MS) in Q Exactive™ plus (Thermo) coupled online to the UPLC. Intact peptides were detected in the Orbitrap at a resolution of 70, 000. Peptides were selected for MS/MS using an NCE setting at 30; ion fragments were detected in the Orbitrap at a resolution of 17,500. A data-dependent procedure that alternated between one MS scan followed by 20 MS/MS scans was applied for the top 20 precursor ions above a threshold ion count of 1E4 in the MS survey scan with 30.0 s dynamic exclusion. The electrospray voltage applied was 2.0 kV. Automatic gain control (AGC) was used to prevent overfilling of the orbitrap; 5E4 ions were accumulated for generation of MS/MS spectra. For MS scans, the m/z scan range was 350 to 1800. Fixed first mass was set as 100 m/z.

### Dataset processing

The protein and acetylation site identification and quantification were performed using Maxquant with an integrated Andromeda search engine (version 1.5.2.8) [28]. Tandem mass spectra were searched against the *Bemisia tabaci* transcriptome data were submitted to the NCBI/SRA database (SRA experiment accession number: SRP056464). Meanwhile, an additional search against *Cardinium* FASTA files downloadable from Uniprot website (http://www.uniprot.org) was also conducted. Trypsin/P was specified as the cleavage enzyme allowing up to 2 missing cleavages, 5 modifications per peptide and 5 charges. Peptide mass tolerance was set at ±20 ppm and fragment mass tolerance at 0.1 Da. Carbamidomethylation on cysteine was specified as fixed modifications and variable modifications were defined as oxidation on methionine and acetylation on lysine. False discovery rate (FDR) thresholds for protein, peptide and modification site were set as a FDR ≤ 1% and minimum peptide length was set at seven. For the quantification method, TMT-6-plex was selected and all other parameters in Maxquant were set to default values.

### Protein annotation

To identify the differently expressed proteins and Kac proteins, A strict fold-change cutoff value of a 1.2-fold or 1/1.2-fold change resulted in differentially expressed proteins compared C^*+^ strain to C^−^ strain. The quantitative ratio over 1.2 was considered as up-regulated proteins while quantitative ratio below 1/1.2 was considered as down-regulated proteins. Comparisons between variables were tested by paired *t* test and *p* values < 0.05 were considered to be statistically significant.

Gene Ontology (GO) annotation analysis was derived from the UniProt-GOA database (www. http://www.ebi.ac.uk/GOA/) [29]. The Kyoto Encyclopedia of Genes and Genomes (KEGG) database was used to identify enriched pathways by a two-tailed Fisher’s exact test to identify the enrichment of the differentially expressed protein and Kac protein against all identified proteins [30]. GO, KEGG pathway and protein domain enrichment were all performed with a corrected *p* < 0.05 using the DAVID bioinformatics resources 6.753 [31]. The functional description of identified proteins were annotated by InterProScan (a sequence analysis application) based on protein sequence alignment using the InterPro domain database [32].

### Motif and secondary structures analysis

Soft motif-x was used to analyze the model of sequences constituted with amino acids in specific positions of modifier-21-mers (10 amino acids upstream and downstream of the site) in all protein sequences [33]. A position-specific heat map was generated by plotting the log10 of the ratio using the heatmap. 2″ function from the “gplots” R-package. Secondary structures were predicted using NetSurfP [34].

### Enrichment-based clustering analysis

All the substrate categories obtained after enrichment were collated, along with their *P* values, and filtered for those categories which were at least enriched in one of the clusters with *P* < 0.01. This filtered *P* value matrix was transformed by the function x = − log10 (*P* value). Finally these x values were z-transformed for each category and the z scores were clustered by one-way hierarchical clustering (Euclidean distance, average linkage clustering) in Genesis. Cluster membership was visualized by a heat map using the “heatmap. 2” function from the “gplots” R-package.

### Protein-protein interaction analysis

All differential expression Kac proteins were blasted to *Acyrthosiphonpisum* species. The search tool for the Retrieval of Interacting Genes/Proteins (STRING) database was used to annotate functional interactions of all the identified acetylated proteins by calculating their confidence score [35]. All interactions that had a confidence score ≥ 0.4 in the STRING database were fetched for the analysis. Cytoscape software (version 3.0.1) was used to visualize the interaction network [36].

## Results

### Overview of the proteome in *Bemisia tabaci*

The proteomes of *B. tabaci* C^*+^ and C^−^ strains were successively performed, including three independent biological replicates for each sample. In total, 3858 proteins were identified, of which 3353 proteins were quantified with a TMT labeling efficiency greater than 99% (Additional file 2: Table S1). A total of 146 differentially expressed proteins containing 77 up-regulated proteins and 69 down-regulated proteins were identified in the C^*+^ compared to C- strains with a high degree of repeatability in the experiment according to the criteria (fold change ratio > 1.2 and *p* < 0.05) (Additional file 2: Table S2).

### Subcellular localization of differently expressed proteins

Bioinformatics analysis on GO and subcellular locations of the differentially expressed proteins in C^*+^ strain compared to C^−^ strain were carried out. GO analysis showed that the 77 up- regulated proteins were mainly sub-categorized into 17 hierarchically-structured GO classifications including 6 biological processes, 6 cellular components, and 5 molecular functions (Additional file 1: Figure S2). For all down- regulated proteins, 14 hierarchically-structured GO classifications including 6 biological processes, 4 cellular components, and 4 molecular functions (Additional file 1: Figure S3; Additional file 2: Table S3). The 77 up-regulated proteins were mainly distributed in cytosol (26%), extracellular (26%) and nuclear (17%). In contrast, the 69 down-regulated proteins were mainly distributed in cytosol (25%), extracellular (23%) and mitochondria (15%) (Additional file 1: Figure S4; Additional file 2: Table S4).

### Enrichment analysis of differentially expressed proteins

To elucidate the functional differences between the down-regulated and up-regulated proteins, the quantified proteins were examined using GO enrichment analysis (Additional file 2: Table S5). In molecular functions category, many of up-regulated proteins were highly enriched in RNA-directed RNA polymerase activity, RNA polymerase activity, cysteine-type peptidase activity, heme binding and terapyrrole binding. These results suggest that the up-regulated proteins might be highly associated with RNA metabolism. The down-regulated proteins were enriched for the following: iron ion binding, phosphatase activity and transition metal ion binding, oxidoreductase activity, heme binding, terapyrrole binding, phosphoric ester hydrolase activity and nucleic acid binding. In cellular components category, the down-regulated proteins were highly enriched in cytoplasm, whereas the up-regulated proteins were no only highly enriched terms. In biological processes category, the down-regulated proteins were highly enriched in DNA metabolic processes, whereas the up-regulated proteins were not enriched in any biological process category.

To further investigate their biological functions, KEGG pathway analysis was performed on differentially expressed proteins in the C^*+^ and C^−^ strains (Additional file 2: Table S6). A total of seven biological pathways were enriched (*P* < 0.05) for up-regulated proteins as follows: p53 signaling pathway, platinum drug resistance, viral myocarditis, apoptosis, glyoxylate and dicarboxylate metabolism, and retinol metabolism. Besides, down-regulated proteins were mainly enriched in only spliceosome.

### Overview of the acetylome in *Bemisia tabaci*

The acetylated proteins and their modification sites were identified using TMT labeling and lysine acetylated (K^ac^) affinity enrichment followed by high-resolution LC-MS/MS in *B. tabaci* C^*+^ strain compared to C^−^ strain. A total of 528 lysine acetylation sites in 283 protein groups were identified, among which 356 sites in 202 proteins were accurately quantified (Additional file 2: Table S7). We subsequently used the quantification results of the global proteome to normalize the acetylome quantification data. From these, 30 sites in 26 lysine acetylation proteins were quantified as up-regulated targets and 35 sites in 29 lysine acetylation proteins were quantified as down-regulated targets at a threshold of 1.2 (*P* < 0.05) (Additional file 2: Table S8). Indeed, as the first lysine acetylome map of *B.tabaci* in response to *Cardinium* infection, it is expected to supply valuable resources for PTM study in the future.

**Fig. 2:**
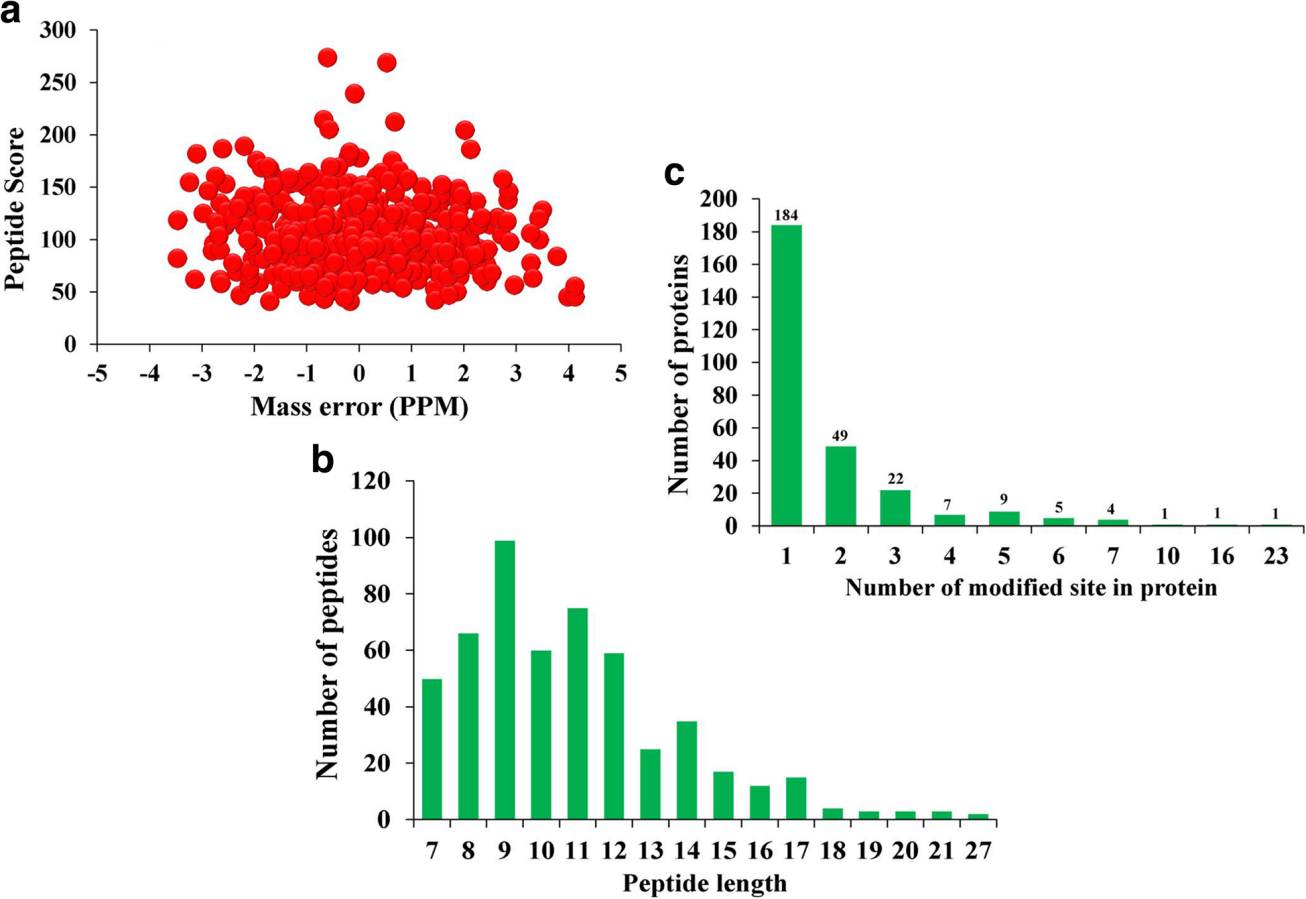
Identification of lysine acetylation in *Bemisia tabaci*. **a** Mass error distribution of all identified peptides. **b** Peptide length distribution. **c** Distribution of acetylated proteins based on their number of acetylation peptides

In the identification, the mass errors were lower than 5 ppm and the most of them were near zero, confirming sample preparation reached the standard (Fig. 2a). The length of most peptides was distributed between 8 and 20, which agreed with the property of tryptic peptides (Fig. 2b). The acetylated proteins contained different numbers of acetylation sites from 1 to 23, and there were 184 acetylated proteins containing only one acetylation site, accounting for 40.0% of total acetylated proteins. The proportion of proteins with two, three, four or more modification sites were 10.4, 4.7 and 4.4%, respectively (Fig. 2c).

### Sequence properties of lysine acetylated proteins in *Bemisia tabaci*

**Fig. 3:**
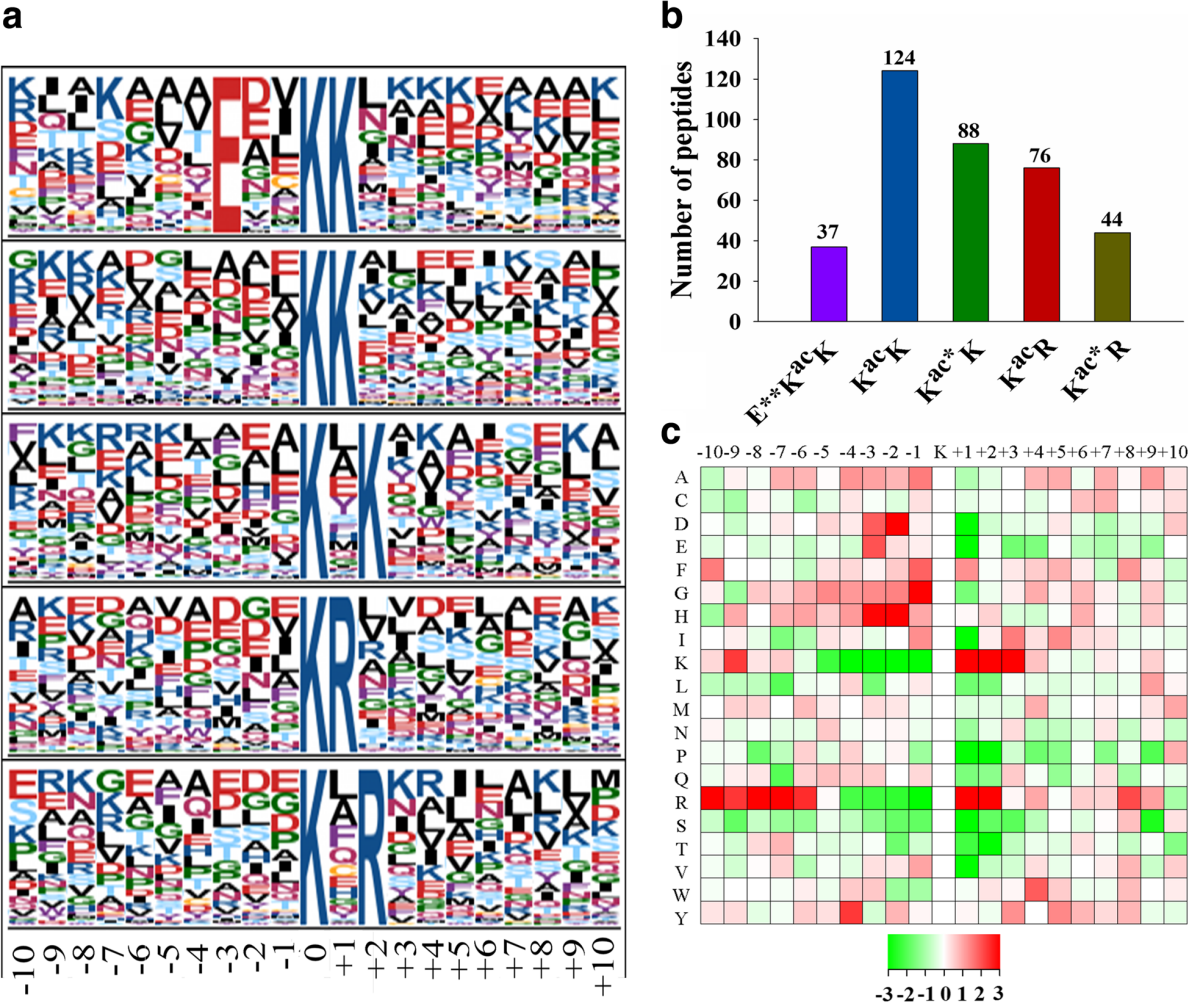
Properties of the acetylated peptides in *Bemisia tabaci*. **a** Acetylation motifs and conservation of acetylation sites. **b** Number of identified modification sites in each acetylated protein. **c** Heat map of the amino acid compositions of the acetylation sites. (Red indicates enrichment and green identifies depletion

To understand the properties of lysine acetylation sites, the occupancy frequency of amino acids in positions surrounding the identified modification sites were examined using Motif-X. A total of 369 peptides (accounting for 69.9% of total peptides identified) included amino acid sequences from position − 10 to + 10 around acetylated lysine in *B. tabaci* (Fig. 3a). These sequences were matched to five conserved motifs near acetylation sites, namely, E**KacK, KacK, Kac*K, KacR and Kac*R, which exhibit different abundances (* represents a random amino acid residue). Motifs KacK, Kac*K and KacR were conserved as peptides and these motifs accounted for approximately 78% of all the identified peptides, providing some insight into *Cardinium* responses in *B. tabaci* (Fig. 3b). With the exception of glutamic acid (E), all enrichment residues arginine (R) and lysine (K) were found downstream of the acetylated lysine. Moreover, as shown in the heat map of amino acid compositions surrounding the acetylation sites, the enrichments of K and R in + 1 to + 2 positions in the motifs were revealed to be the highest, which can infer that proteins with such motifs in these positions are preferred substrates of lysine acetytransferases in the cell (Fig. 3c; Additional file 2: Table S9).

### Function analysis of differentially expressed Kac proteins

**Fig. 4:**
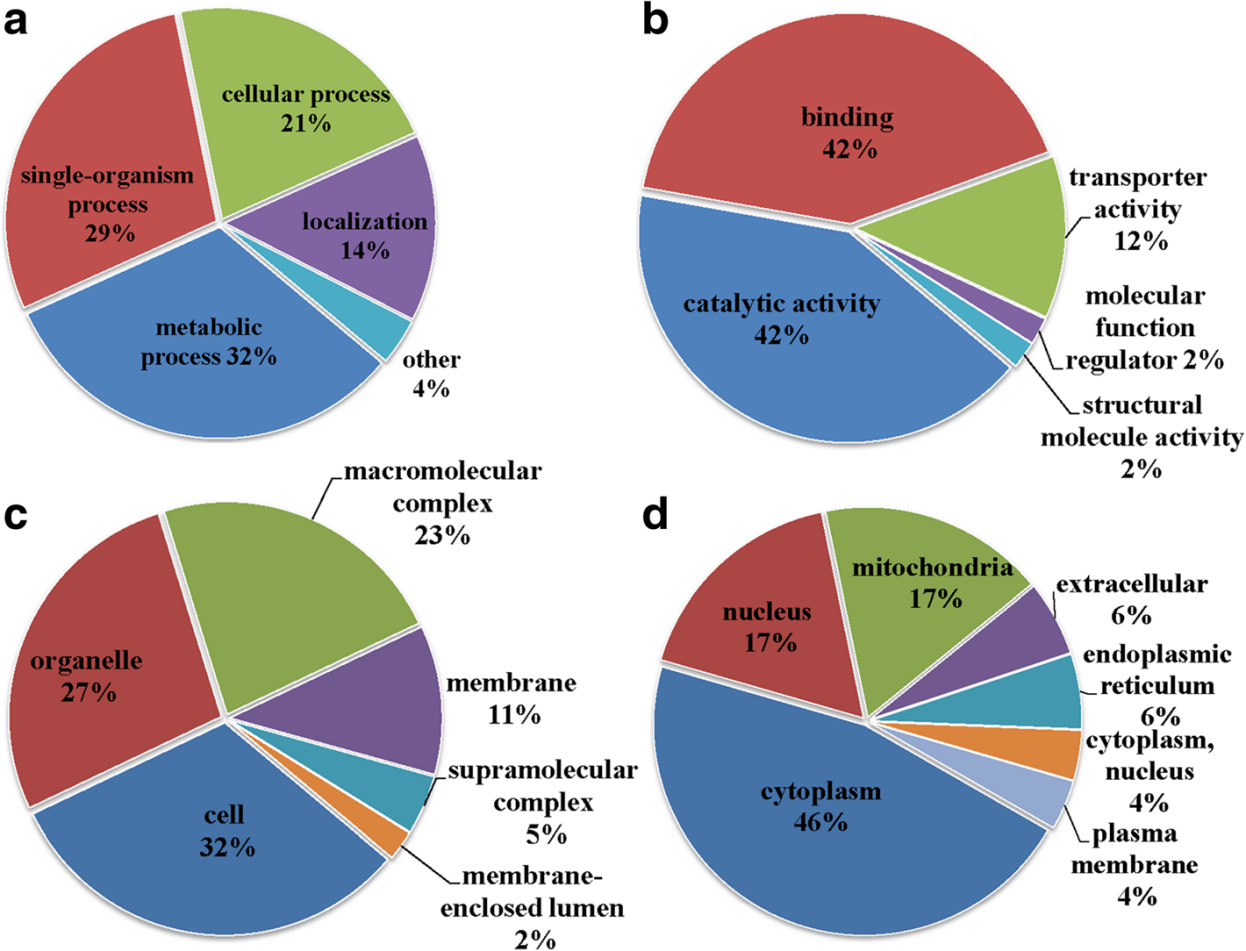
Functional classification of differentially expressed Kac proteins compared C^*+^ to C^−^ strains. **a** Classification of the differentially expressed Kac proteins based on biological process. **b** Classification of the differentially expressed lysine acetylated proteins based on molecular function. **c** Classification of the differentially expressed lysine acetylated proteins based on cellular component. **d** Subcellular localization of the differentially expressed lysine acetylated proteins

To characterize the functions and subcellular locations of differentially expressed Kac proteins in response to *Cardinium* infection in *B. tabaci*, GO functional classification and subcellular functional annotation were performed (Fig. 4; Additional file 2: Table S10). There were three larger protein groups of differentially expressed Kac proteins involved in metabolic processes (32%), single- organism process (29%) and cellular processes (21%). According to the molecular function classification, most differentially expressed Kac proteins were found to be related to catalytic activity (42%) and binding (42%). The results of cellular component analysis revealed that the differentially expressed Kac proteins were catalogued in the cell (32%), organelles (27%), macromolecular complexes (23%) and membranes (11%). These results demonstrate that the differentially expressed Kac proteins referring to the cellular component, with diversified molecular functions, are involved in a variety of biological processes. Subcellular distribution predictions showed that differentially expressed Kac proteins distributed predominantly in cytosol (46%) and the mitochondria (17%); nuclear (17%) was also a highly represented term in the acetylome. Significantly, three differentially expressed Kac proteins (6%) were found to be distributed in the extracellular space, highlighting that differentially expressed Kac proteins have multiple functions.

**Fig. 5:**
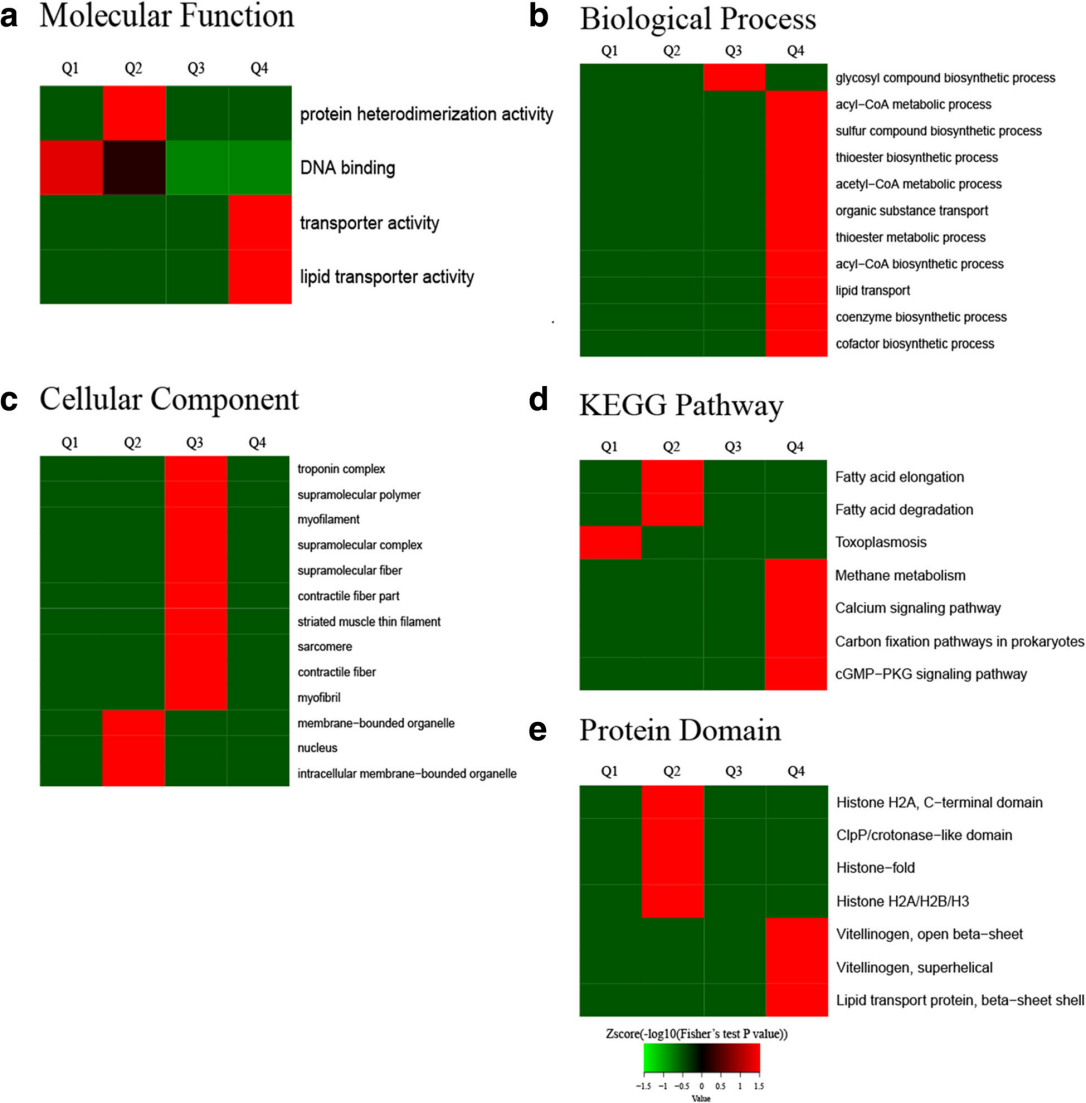
Enrichment-based clustering analysis of acetylome data sets in *Bemisia tabaci* C^*+^ and C^−^ strains. **a** molecular function, **b** biological process, **c** cellular compartment, **d** KEGG pathways, **e** protein domain. In each classification, all the quantified proteins were divided into four groups according to C^*+^/C- ratios: Q1 (Ratio < 0.77), Q2 (0.77 < Ratio < 0.83), Q3 (1.2 < Ratio < 1.3), Q4 (Ratio > 1.3)

To gain greater insight into the preferred functional enrichment of corresponding *Cardinium*- responsive Kac proteins, three types of enrichment-based clustering analyses were performed: GO, KEGG pathway, protein domain analysis (Fig. 5). All the quantified Kac proteins were divided into four quantiles according to the C^*+^/C- ratio to generate four quantiles: Q1 (Ratio < 0.77), Q2 (0.77 < Ratio < 0.83), Q3 (1.2 < Ratio < 1.3), and Q4 (Ratio > 1.3). Enrichment analyses were performed separately based on the quantiles.

In GO functional enrichment (Fig. 5a–c; Additional file 2: Table S11), the analysis of molecular function (Fig. 5a) showed that the down-regulated Kac proteins participated in DNA binding and protein heterodimerization, and up-regulated Kac proteins involved in lipid transporter activity and transporter activity. In biological process (Fig. 5b), the up-regulated Kac proteins were mostly enriched in lipid transport, coenzyme biosynthetic process, acyl-CoA metabolic process, thioester metabolic process and other process, suggesting the metabolisms and biosynthesis of proteins may be regulated by lysine acetylation. However, down-regulated proteins showed no enriched terms. In the cellular component category (Fig. 5c), up-regulated proteins were mainly distributed in myofibril, troponin complex, striated muscle thin filament and supramolecular complex. In contrast, down-regulated proteins were located on membrane-bound organelle, nucleus and intracellular membrane-bound organelle, which may be of considerable interest as potential drug targets.

To identify cellular pathways of *B. tabaci* infected with *Cardinium*, we then performed a pathway clustering analysis using KEGG (Fig. 5d; Additional file 2: Table S12). According to KEGG annotation, the proteins belonging to the categories for metabolism were mainly identified as Kac proteins. The results showed that methane metabolism, calcium signaling pathway, carbon fixation pathways in prokaryotes and cGMP-PKG signaling pathway were mostly enriched for up-regulated Kac proteins. Besides, the pathway termed toxoplasmosis, fatty acid elongation and fatty acid degradation enriched by down-regulated Kac proteins.

As domain structure is a critically important functional feature of proteins, we next analyzed this domain after *B. tabaci* was infected with *Cardinium* (Fig. 5e; Additional file 2: Table S13). We found that domains in proteins involved in Histone-fold, Histon eH2A C-terminal domain HistoneH2A/H2B/H3, ClpP/cotonase-like domin were remarkably enriched in down-regulated Kac proteins. However, the vitellinogen, open beta-sheet, superhelical and lipid transport protein, were mainly enriched in up-regulated Kac proteins.

### Protein interaction networks of differentially expressed Kac proteins

**Fig. 6:**
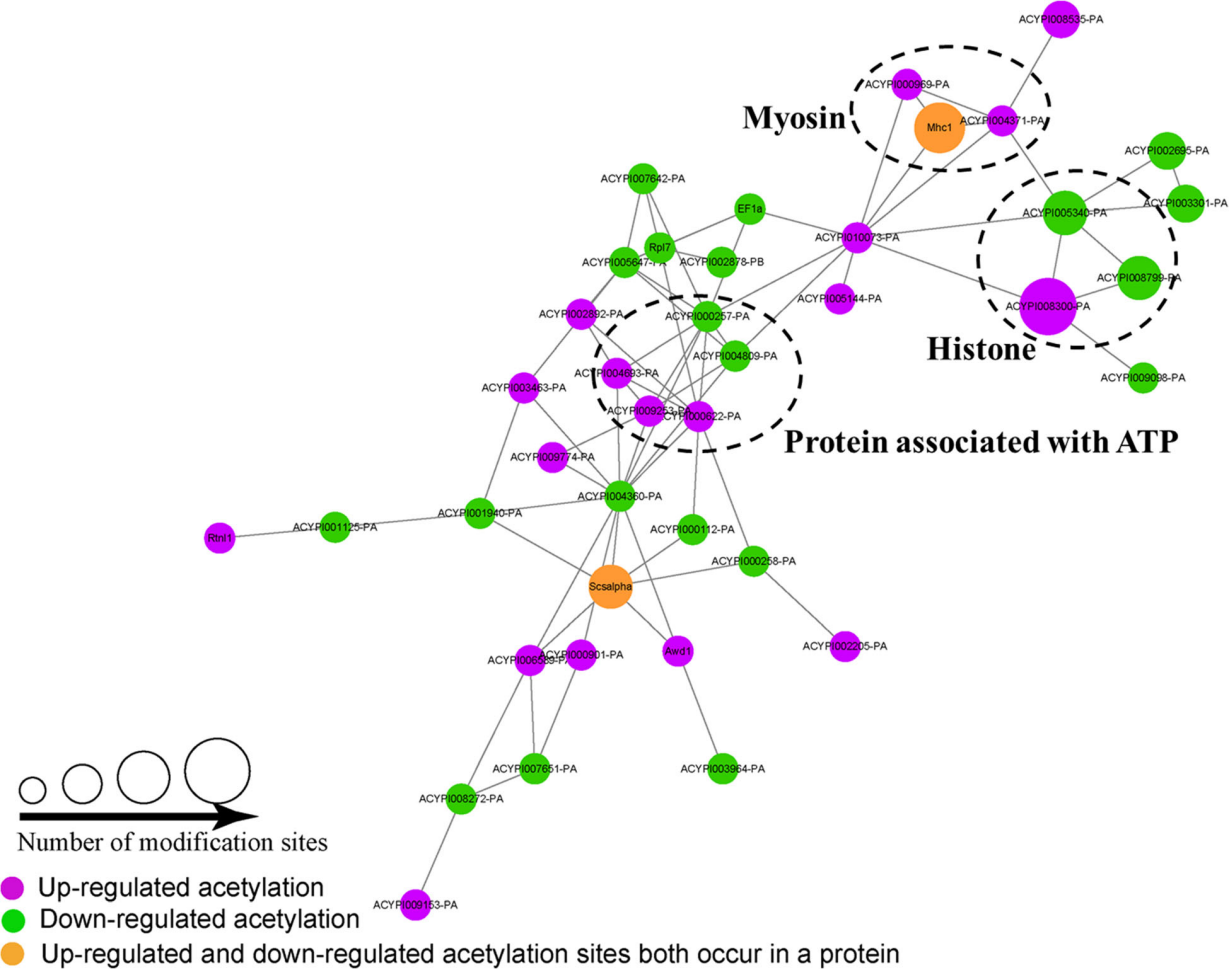
Interaction networks of differentially expressed Kac proteins compared *Bemisia tabaci* C^*+^ to C^−^ strains

Protein-protein interaction network analysis was conducted to disclose the important nodes and crucial interactions among the differentially expressed Kac proteins. The results showed that a total of 40 differentially expressed Kac proteins were mapped to the protein interaction database. We observed tight communications among the differentially expressed Kac proteins and highly connected clusters in histone, myosin and protein associated with ATP (Fig. 6; Additional file 2: Table S14).

## Discussion

Although lysine acetylation is a widespread and highly conserved post-translational modification in both eukaryotes and prokaryotes with diverse biological functions [37], little is known about the function of this modification in *B. tabaci*, in particular in response to *Cardinium* infection. In this study, we determined the proteomes and acetylomes of *B. tabaci* Q in response to *Cardinium* infection using the combination of affinity enrichment and high-resolution LC-MS/MS analysis. Intensive bioinformatics analysis showed that differentially expressed proteins and Kac proteins are widely distributed and participate in diverse biological processes. Moreover, protein interaction network analyses demonstrated widespread interactions modulated by protein acetylation. These data will be expected to supply important resource for exploring the arthropod-symbiont interaction.

As functional analysis of differentially expressed proteins compared C^*+^ to C^−^ strains, we found that up-regulated proteins mainly enriched in seven biological pathways including p53 signaling pathway, platinum drug resistance, viral myocarditis, apoptosis, glyoxylate and dicarboxylate metabolism, and retinol metabolism. Retinol metabolism is of crucial importance for many physiological processes, including embryonic development, reproduction, postnatal growth, differentiation and maintenance of various epithelia, immune responses and vision [38, 39]. We therefore speculate that these up-regulated proteins may play important roles in immune responses of *B. tabaci* Q to *Cardinium* infection. Besides, down-regulated proteins were mainly enriched in spliceosome, suggesting that down-regulated proteins associated with the spliceosome may change the alternative pre-mRNA splicing that take place during *Cardinium* infection in *B. tabaci*.

In this study, a total of 528 lysine acetylation sites in 283 protein groups were identified, among which 356 sites in 202 proteins were accurately quantified. Probably due to the limited genome information in *B. tabaci*, the numbers of detected acetylation sites and proteins are much less than those in *Drosophila* with well assembled whole genome sequences [23]. Besides, five conserved motifs near acetylation sites named as E**KacK, KacK, Kac*K, KacR and Kac*R exhibit different abundances. Among these motifs, alkalescent amino acid (R and K) was observed in the + 1 and + 2 positions, which were rarely identified in other organisms [22, 24, 40, 41], suggesting they may be functionally important for acetylation to occur in *B. tabaci*. Moreover, the heat map of amino acid compositions surrounding the acetylation sites showed the frequency of K and R in + 1 to + 2 positions in the motifs is the highest. The result is inconsistent with previous studies [41, 42] which revealed the frequency of K and R in positions − 2 to − 1 in the motifs is the lowest.

As GO enrichment analysis shown, the up-regulated Kac proteins were mainly involved in lipid transporter activity and transporter activity, which is consistent with a previous research suggesting lipid transporter activity may be regulated by lysine acetylation in silkworm [22]. Among the KEGG pathway analysis, we found that methane metabolism, calcium signaling pathway, carbon fixation pathways in prokaryotes and cGMP-PKG signaling pathway were mostly enriched for up-regulated Kac proteins. The methane metabolism pathways were related to energy metabolism, suggesting these Kac proteins may be preferably targeting the energy metabolism. The pathway termed toxoplasmosis, fatty acid elongation and fatty acid degradation enriched by down-regulated Kac proteins were associated with infectious diseases and lipid metabolism [43, 44]. Many studies have revealed that toxoplasmosis in early life might affect neurodevelopment and contribute to later onset of schizophrenia [45–47]. Thus, we speculated that *Cardinium* infection may lead to serious physiological disorder in *B. tabaci* Q.

Besides, domain structure analysis showed that *Cardinium* infection negatively regulates the acelytation of histones and may play critical roles in regulating many processes within the nucleus, including transcription initiation and elongation, silencing, and DNA repair, by decreasing the acetylation levels of histones in *B. tabaci* [48]. Furthermore, protein-protein interaction network analysis demonstrated that differentially expressed Kac proteins and highly connected clusters in histone, myosin and protein associated with ATP. Previous studies have revealed that a highly enriched domain in Kac proteins was the myosin tail domain. Myosin is responsible for actin-based motility, suggesting another role of acetylation in muscle contraction and motility processes in silkworms [22]. The interaction networks suggest that the Kac proteins involve in nucleus processes, motility processes and energy metabolism. Indeed, metabolic process is the primary biological process involving acetylation in many species, such as *Escherichia coli*, *S. roseosporus* and *Vibrio parahemolyticus* [26, 49, 50], suggesting that metabolism may be comprehensively regulated by lysine acetylation in *B. tabaci*.

## Conclusions

In this study, we identified a total of 146 differentially expressed proteins and 30 sites in 26 lysine acetylation proteins were quantified as up-regulated targets and 35 sites in 29 lysine acetylation proteins were quantified as down-regulated targets. These differentially expressed proteins and lysine acetylated proteins were mainly involved in an extensive range of biological processes and metabolic pathways, indicating the wide regulation of *Cardinium* on the proteome and acetylome of *B. tabaci* Q. This study widens the range of physiological processes regulated by lysine acetylation and provides a rich resource for exploring the functions of acetylation in arthropod-symbiont interaction.

## Additional files

Additional file 1: **Figure S1.** Amplification product of *Cardinium*-infected *Bemisia tabaci* Q using specific primers. 1–10: *Cardinium*-infected *B. tabaci* Q; +: positive control; −: negative control; M: DNA marker. **Figure S2.** The subcellular location of up- (A) and down-(B) regulated proteins compared C^*+^ to C^−^ strains. **Figure S3.** GO distribution of up-regulated proteins in biological process (A), cellular component (B) and molecular function terms (C) compared C^*+^ to C^−^ strains. **Figure S4.** GO classification of down-regulated proteins in biological process (A), cellular component (B) and molecular function terms (C) compared C^*+^ to C^−^ strains. (ZIP 158 kb)

Additional file 2: **Table S1.** List of proteins identified and quantified by the TMT analysis in the experiment. **Table S2.** List of differential expressed proteins by the TMT analysis in the experiment. **Table S3.** Subcellular location of differentially expressed proteins compared C^*+^ to C^−^ strains. **Table S4.** Gene Ontology classification of differentially expressed proteins compared C^*+^ to C^−^ strains. **Table S5.** GO annotation of differentially expressed proteins compared C^*+^ to C^−^ strains. **Table S6.** KEGG pathway of differentially expressed proteins compared C^*+^ to C^−^ strains. **Table S7.** Detailed information on identified acetylated peptides compared C^*+^ to C^−^ strains. **Table S8.** Detailed information of differentially expressed acetylation peptides compared C^*+^ to C^−^ strains. **Table S9.** Amino acid sequence analysis from the − 10 to + 10 positions around the acetylated lysine. **Table S10.** GO functional classification of differentially expressed acetylated proteins compared C^*+^ to C^−^ strains. **Table S11.** GO enrichment analysis of differentially expressed lysine acetylated proteins using Blast2GO. **Table S12.** KEGG pathway enrichment analysis of differentially expressed acetylated proteins compared C^*+^ to C^−^ strains. **Table S13.** Domain enrichment analysis of differentially expressed acetylated proteins compared C^*+^ to C^−^ strains. **Table S14.** Interaction networks of differentially expressed Kac proteins compared C^*+^ to C^−^ strains. (ZIP 953 kb)

## Abbreviations

AGC: Automatic gain control
C^−^: *Cardinium*-uninfected strain
C^*+^: *Cardinium*-infected *B. tabaci* Q strain
CAN: Acetonitrile
FA: Formic acid
Kac: Acetylated lysine
NSI: Nano electrospray ionization
PTM: Post-translational modification
TMT: Tandem mass tagging
