# Supplementary figures and images for: Novel proteome and acetylome of Bemisia tabaci Q in response to Cardinium infection
Source: BMC Genomics. 2018 Jul 5;19:523. doi: 10.1186/s12864-018-4907-3 (PMC6034306; doi:10.1186/s12864-018-4907-3)

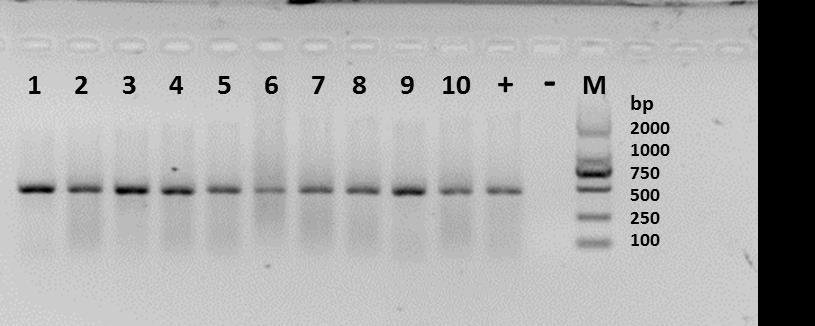

Supplement: Supplementary file 1 — Figure S1. Amplification product of Cardinium-infected Bemisia tabaci Q using specific primers. 1–10: Cardinium-infected B. tabaci Q; +: positive control; −: negative control; M: DNA marker. Figure S2. The subcellular location of up- (A) and down-(B) regulated proteins compared C*+ to C− strains. Figure S3. GO distribution of up-regulated proteins in biological process (A), cellular component (B) and molecular function terms (C) compared C*+ to C− strains. Figure S4. GO classification of down-regulated proteins in biological process (A), cellular component (B) and molecular function terms (C) compared C*+ to C− strains. (ZIP 158 kb) [file 12864_2018_4907_MOESM1_ESM.zip › FIgure S1.jpg]

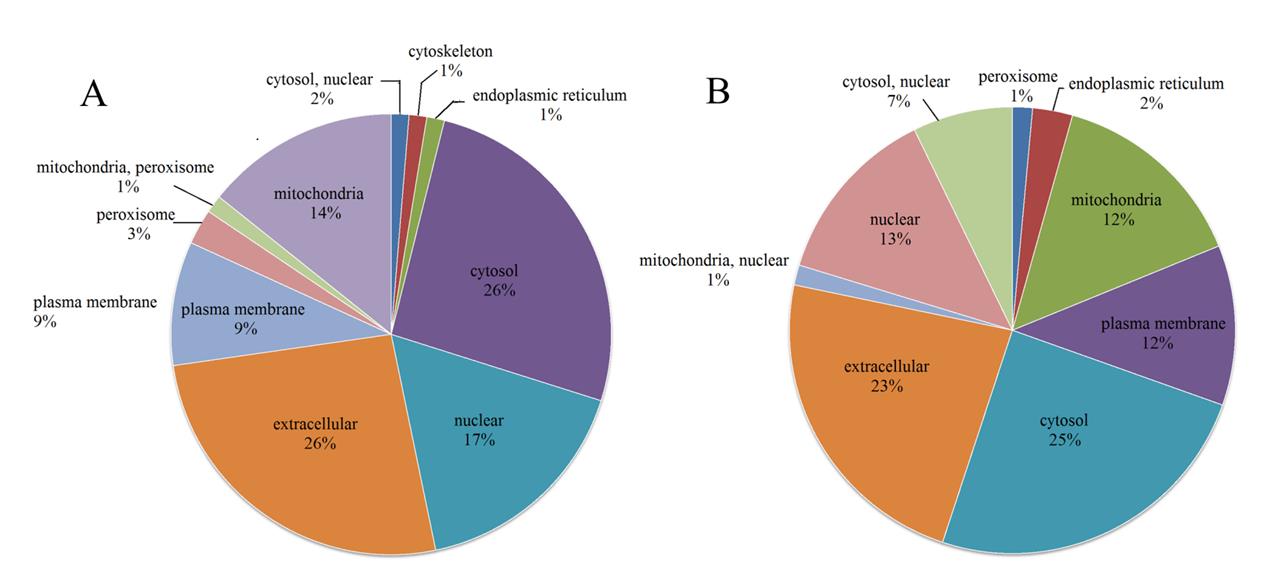

Supplement: Supplementary file 1 — Figure S1. Amplification product of Cardinium-infected Bemisia tabaci Q using specific primers. 1–10: Cardinium-infected B. tabaci Q; +: positive control; −: negative control; M: DNA marker. Figure S2. The subcellular location of up- (A) and down-(B) regulated proteins compared C*+ to C− strains. Figure S3. GO distribution of up-regulated proteins in biological process (A), cellular component (B) and molecular function terms (C) compared C*+ to C− strains. Figure S4. GO classification of down-regulated proteins in biological process (A), cellular component (B) and molecular function terms (C) compared C*+ to C− strains. (ZIP 158 kb) [file 12864_2018_4907_MOESM1_ESM.zip › FIgure S2.jpg]

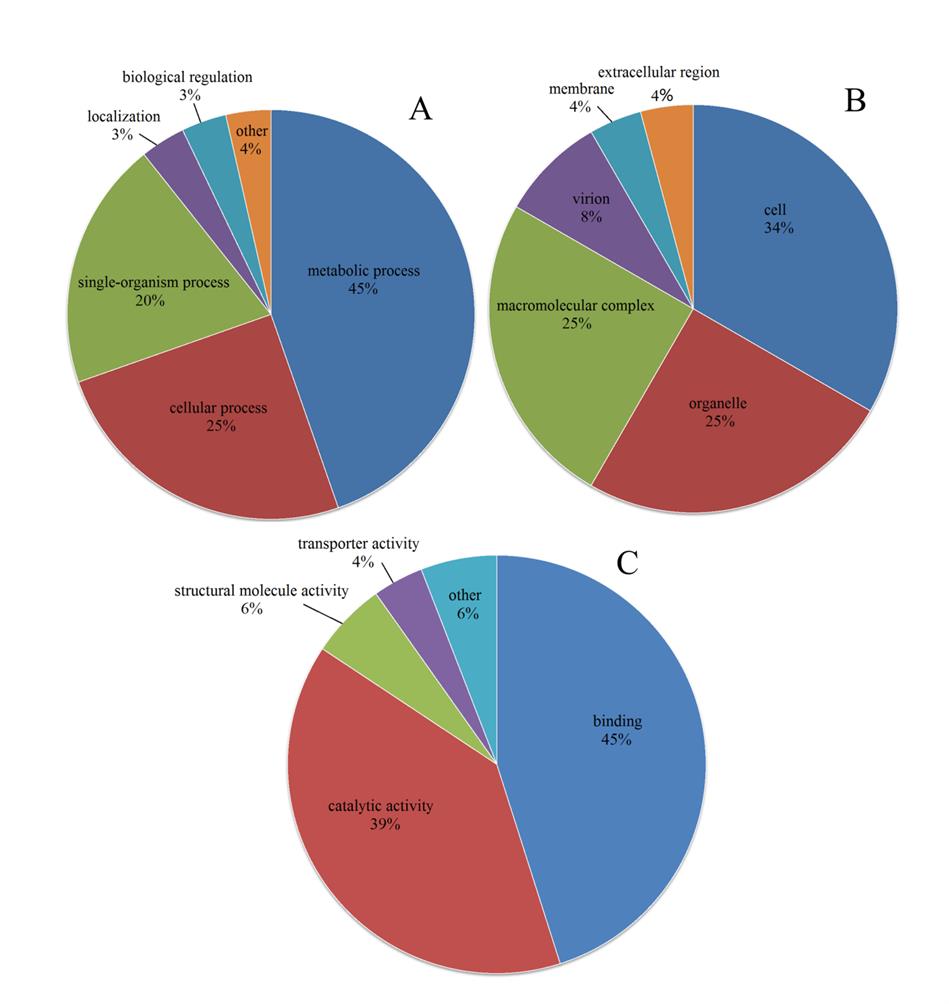

Supplement: Supplementary file 1 — Figure S1. Amplification product of Cardinium-infected Bemisia tabaci Q using specific primers. 1–10: Cardinium-infected B. tabaci Q; +: positive control; −: negative control; M: DNA marker. Figure S2. The subcellular location of up- (A) and down-(B) regulated proteins compared C*+ to C− strains. Figure S3. GO distribution of up-regulated proteins in biological process (A), cellular component (B) and molecular function terms (C) compared C*+ to C− strains. Figure S4. GO classification of down-regulated proteins in biological process (A), cellular component (B) and molecular function terms (C) compared C*+ to C− strains. (ZIP 158 kb) [file 12864_2018_4907_MOESM1_ESM.zip › FIgure S3.jpg]

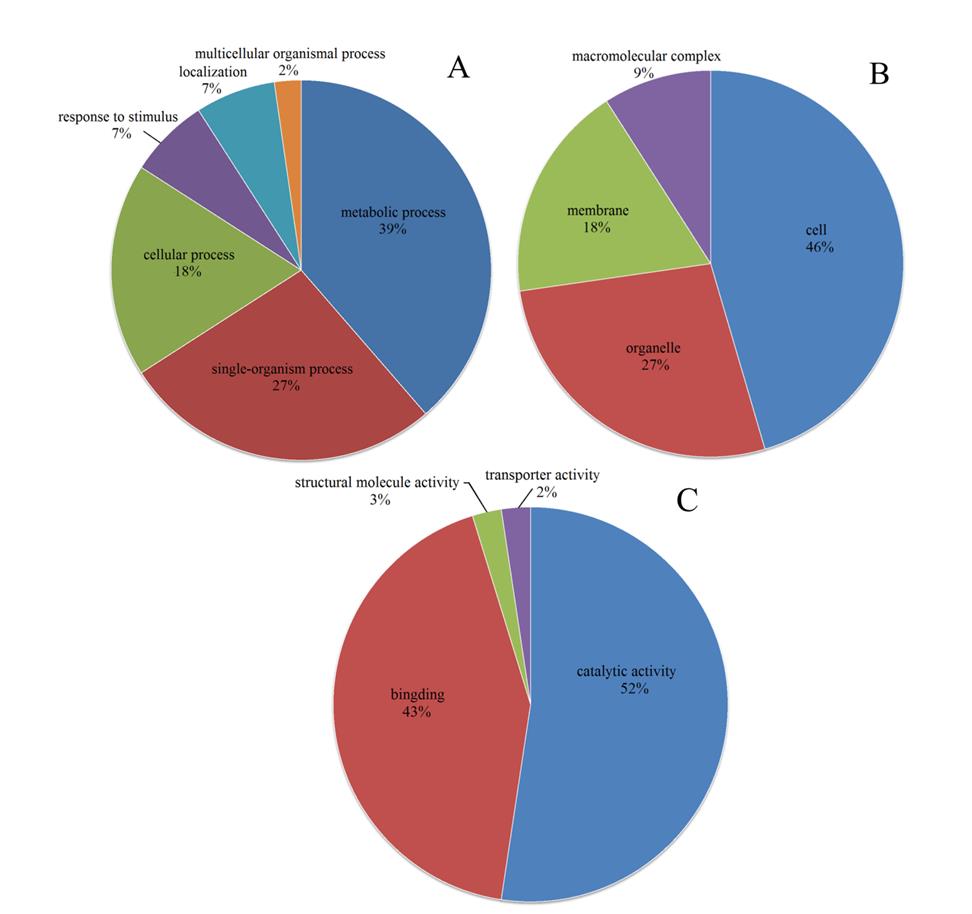

Supplement: Supplementary file 1 — Figure S1. Amplification product of Cardinium-infected Bemisia tabaci Q using specific primers. 1–10: Cardinium-infected B. tabaci Q; +: positive control; −: negative control; M: DNA marker. Figure S2. The subcellular location of up- (A) and down-(B) regulated proteins compared C*+ to C− strains. Figure S3. GO distribution of up-regulated proteins in biological process (A), cellular component (B) and molecular function terms (C) compared C*+ to C− strains. Figure S4. GO classification of down-regulated proteins in biological process (A), cellular component (B) and molecular function terms (C) compared C*+ to C− strains. (ZIP 158 kb) [file 12864_2018_4907_MOESM1_ESM.zip › FIgure S4.jpg]
